# Pre-existing conditions are associated with COVID-19 patients’ hospitalization, despite confirmed clearance of SARS-CoV-2 virus

**DOI:** 10.1016/j.eclinm.2021.100793

**Published:** 2021-03-23

**Authors:** Colin Pawlowski, AJ Venkatakrishnan, Eshwan Ramudu, Christian Kirkup, Arjun Puranik, Nikhil Kayal, Gabriela Berner, Akash Anand, Rakesh Barve, John C. O'Horo, Andrew D. Badley, Venky Soundararajan

**Affiliations:** a nference, One Main Street, Cambridge, MA 02142, United States; b Mayo Clinic, Rochester, MN 55902, United States; c nference Labs, Indiqube, 22, 3rd Cross Rd, S R Layout, Murgesh Pallya, Bengaluru, Karnataka 560017, India

## Abstract

**Background:**

Consecutive negative SARS-CoV-2 PCR test results are being considered to estimate viral clearance in COVID-19 patients. However, there are anecdotal reports of hospitalization from protracted COVID-19 complications despite such confirmed viral clearance, presenting a clinical conundrum.

**Methods:**

We conducted a retrospective analysis of 222 hospitalized COVID-19 patients to compare those that were readmitted post-viral clearance (*hospitalized post-clearance cohort, n* = 49) with those that were not re-admitted post-viral clearance (*non-hospitalized post-clearance cohort, n* = 173) between February and October 2020. In order to differentiate these two cohorts, we used neural network models for the ‘augmented curation’ of comorbidities and complications with positive sentiment in the Electronic Hosptial Records physician notes.

**Findings:**

In the year preceding COVID-19 onset, anemia (*n* = 13 [26.5%], p-value: 0.007), cardiac arrhythmias (*n* = 14 [28.6%], p-value: 0.015), and acute kidney injury (*n* = 7 [14.3%], p-value: 0.030) were significantly enriched in the physician notes of the *hospitalized post-clearance cohort*.

**Interpretation:**

Overall, this retrospective study highlights specific pre-existing conditions that are associated with higher hospitalization rates in COVID-19 patients despite viral clearance and motivates follow-up prospective research into the associated risk factors.

**Funding:**

This work was supported by Nference, inc.

**Research in Context**

**Evidence before this study**

There are several case series reports that suggest that individuals infected with SARS-CoV-2 may continue to experience symptoms weeks or months after their initial infection.

**Added value of this study**

This study provides a comparison of 222 hospitalized COVID-19 patients that were re-admitted vs. not re-admitted to the hospital following confirmed viral clearance of SARS-CoV-2, as determined via two or more consecutive negative PCR tests. We find that in the year preceding COVID-19, acute kidney injury, anemia, and cardiac arrhythmia were significantly enriched in the physician notes of the hospitalized post-clearance cohort.

**Implications of all the available evidence**

This study shows that hospitalized COVID-19 patients who were re-admitted to the hospital following confirmed viral clearance of SARS-CoV-2 had higher rates of pre-existing conditions including: acute kidney injury, anemia, and cardiac arrhythmias. This suggests that these pre-existing conditions may be risk factors for the post-clearance complications of COVID-19 which require hospitalization.

Alt-text: Unlabelled box

## 1 Introduction

To date, over 103 million people worldwide have been infected with SARS-CoV-2, with nearly 2.23 million deaths as of December 2020 [1]. At this point in the pandemic, there are reports that some patients who have cleared the virus have tested positive after documented negative tests or have developed new symptoms requiring hospitalization after documented negative tests [2], [3], [4], [5], [6]. A recent case series [7] reported four patients with apparent SARS-CoV-2 reinfection after an index hospitalization, despite resolution of symptoms and radiographic abnormalities and two consecutive negative tests separated by a day. A single center cohort study of 414 patients with SARS-CoV-2 reported a reinfection rate of 16.7% among cleared patients [5], while a second cohort study of 262 patients with SARS-CoV-2 reported a reinfection rate of 14.5% among cleared patients [4].

Further, probing the comorbidities associated with pre and post SARS-CoV-2 infection is a comprehensive approach to reveal the biological phenomenon causing varying disease severity levels across COVID-19 patients. Accordingly, many meta-analyses identified that individuals with pre-existing disease conditions such as cancer [8], cerebrovascular disease [9], type 2 diabetes mellitus [10], chronic obstructive pulmonary disease (COPD) [11,12], hypertension [12,13], and chronic kidney disease [14,15] are more susceptible to COVID-19 infection. Likewise, a few post-SARS-CoV-2 clearance follow-up studies (3–6 months) have reported chronic fatigue, mental illness, breathlessness and insomnia being the most common disorders observed in the discharged patients [16,17]. However, the details on the outcomes of patients who require hospitalization after documented clearance of SARS-CoV-2 remain unknown. These hospitalizations can have enormous implications beyond individual outcomes in shaping how we guide our public health strategy, allocate healthcare resources, and identify those at greatest risk.

Comparing the patient records of hospitalized patients that are readmitted post-viral clearance with a control group of patients that were not re-admitted post-viral clearance will enable us to identify whether there are pre-existing conditions that are associated with COVID-19 patients’ hospitalization, despite confirmed clearance of SARS-CoV-2 virus. Here, we refer to this study cohort as the *hospitalized post-clearance cohort*, and we refer to this control group as the *non-hospitalized post-clearance cohort*. The availability of clinical covariates and outcomes of patients at Mayo Clinic sites and associated health systems provides us an opportunity to perform this analysis. In order to differentiate the *hospitalized post-clearance cohort* and the *non-hospitalized post-clearance cohort*, we use neural network models for the ‘augmented curation’ [18] of pre-existing conditions and complications from the EHR physician notes. We perform the comparisons over the following time horizons: (1) Pre-COVID-19: Day −365 to Day −11 relative to the first positive PCR test for SARS-CoV-2, (2) SARS-CoV-2 positive: Day −10 relative to the first positive PCR test up to the estimated viral clearance date, and (3) Post viral clearance: Day 1 to Day 90 relative to the estimated viral clearance date.

## 2 Methods

### 2.1 Institutional review board (IRB)

This retrospective research was conducted under IRB 20–003,278, ‘Study of COVID-19 patient characteristics with augmented curation of Electronic Health Records (EHR) to inform strategic and operational decisions’. Under this IRB, all the authors had access to the EHR records for all Mayo Clinic patients that were tested using a PCR based method for SARS-CoV-2. The study was deemed exempt by the Mayo Clinic institutional review board and waived from consent. Subjects without research authorization on file were excluded. For further information regarding the Mayo Clinic Institutional Review Board (IRB) policy, and its institutional commitment, membership requirements, review of research, informed consent, recruitment, vulnerable population protection, biologics, and confidentiality policy, please refer to www.mayo.edu/research/institutional-review-board/overview.

### 2.2 Patient and public involvement

The development of the research question and outcome measures was informed by prior literature and information from the Centers for Disease Control and Prevention (CDC) on risk factors for severe COVID-19 illness [19]. No patients were involved in the design of the study, but physicians from the Mayo Clinic who are involved with the COVID-19 research taskforce and the clinical care for COVID-19 patients were involved with the study design and execution. Individuals that opted out of participation in retrospective research studies were not included in this analysis.

### 2.3 Study design

**Fig. 1:**
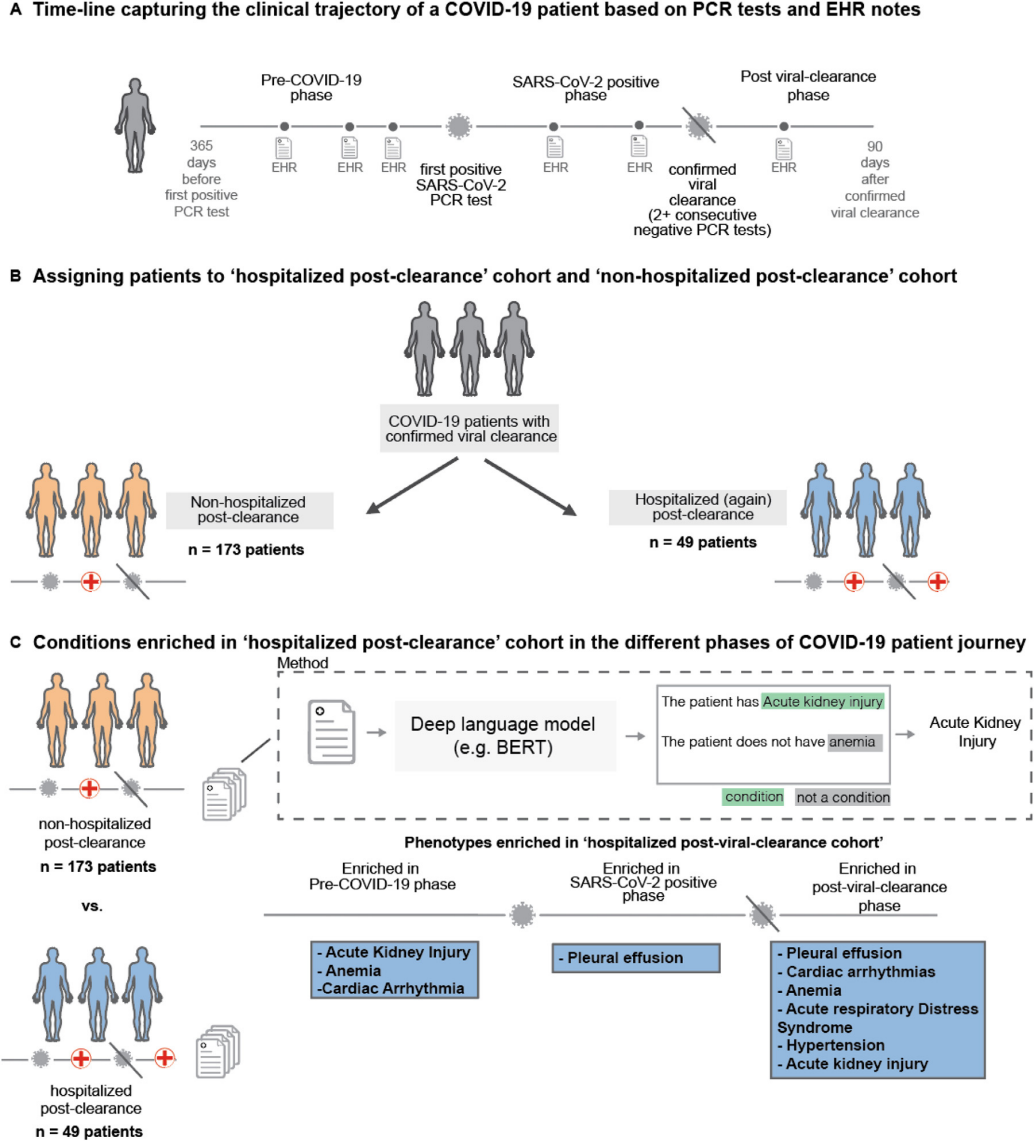
**Overall study design. (A)** Trajectory of a COVID-19 patient transitioning from pre-COVID-19 (time up to 365 days before first positive PCR test) through the SARS-CoV-2 positive phase (interval after first positive test but before the first of two consecutive negative PCR test results) into the post viral clearance phase (period up to 90 days after the first of two negative PCR test results), **(B)** Demonstrates procedure for assigning patients to the hospitalized post-clearance cohort and non-hospitalized post-clearance cohort patients can be hospitalized at varying points in time including during the index infection (time from first positive PCR test results to first of two negative PCR test results) and following viral clearance - two cohorts are defined from the overall population, Hospitalized Post-Clearance Cohort in which patients are admitted or readmitted to the hospital following their estimated clearance date and Non-Hospitalized Post-Clearance Cohort in which patients are admitted during the index infection, but not following the estimated clearance date, **(C)** For each patient if the two defined cohorts a deep language (BERT) model is used to extracted phenotypes of interest from the clinical notes recorded between 365 days prior to infection and up to 90 days after clearance for each patient - occurrences of these phenotypes are stratified into pre-COVID-19, COVID-19 pre-clearance, and COVID-19 post-clearance time periods and statistical tests are run to find significant differences in phenotypes between the two cohorts.

This is an observational study in a cohort of individuals who underwent polymerase chain reaction testing (PCR) for suspected SARS-CoV-2 infection at the Mayo Clinic and hospitals affiliated to the Mayo health system after February 15, 2020. Of 22,223 patients with at least one positive SARS-CoV-2 PCR test, 1355 had two documented negative tests following their last positive test result with an estimated viral clearance date (date of the first negative test) more than 90 days prior to the date of this analysis (October 27, 2020). Among these 1355 patients, 222 patients were admitted to the hospital prior to their estimated viral clearance date, and among these patients, 49 patients were readmitted to the hospital following the estimated viral clearance date. We define the ***hospitalized post-clearance*** cohort to consist of these 49 patients. We note that this cohort does not include patients who simply remained in the hospital following their estimated clearance date. On the other hand, 173 patients were admitted to the hospital pre-clearance, but were not readmitted to the hospital following the estimated clearance date. We refer to this group of 173 patients as the ***non-hospitalized post-clearance*** cohort. The EHR physician notes of the patients were analyzed for both cohorts. All authors had access to the EHR physician notes under IRB during the study period. In Fig. 1, we provide a schematic of the study design and the key findings from this study. In Table 1, we provide the general clinical characteristics for the two cohorts, such as the demographics, COVID-19 pre-clearance hospitalization status, relative clearance date, and comorbidities. The demographics give the age, sex, race, and ethnicity information related to each patient. The COVID-19 pre-clearance hospitalization status indicates whether the patient was admitted to the hospital during the index COVID-19 infection. Next, the relative clearance date provides the number of days between the first positive PCR test and two negative PCR tests. Last, the comorbidities mainly focus on asthma, cancer, chronic kidney disease, chronic obstructive pulmonary disease, obesity, obstructive sleep apnea, type 1 diabetes mellitus, and type 2 diabetes mellitus.

**Table 1:** **General characteristics of study population (patients who “cleared” after previously being SARS-CoV-2 positive) for hospitalized and non-hospitalized post-clearance cohorts.** Demographic variables and comorbidities in the hospitalized and non-hospitalized post-clearance cohorts. Frequency and proportions (n (%)) are shown for the categorical variables, and mean, median, standard deviation, and interquartile range (IQR) are shown for the continuous variables. The columns are: **(1) Hospitalized post-clearance:** clinical characteristics of the cohort of patients who are admitted or readmitted to the hospital following the estimated clearance date of SARS-CoV-2 infection, **(2) Non-Hospitalized post-clearance:** clinical characteristics of the control group of patients who are not admitted to the hospital following the estimated clearance date, **(3) BH-corrected p-value:** Benjamani-Hochberg corrected p-values using Fisher's exact test for comparisons of proportions and Mann-Whitney U test for continuous covariates.

Clinical covariate	Hospitalized Post-Clearance	Non-Hospitalized Post-Clearance	BH-adjusted p-value
Total number of patients	49	173	
Age in years - Mean - Median - Standard deviation - IQR	58.9 61.8 18.5 (47.6, 72.5)	58.5 58.9 16.7 (49.4, 71.6)	0.959
Relative Cleared Date in days (standard deviation) - Mean - Median - Standard deviation - IQR	21.7 20.0 13.5 (14.0, 31.0)	21.7 20.0 12.8 (14.0, 29.0)	0.993
Sex - Female - Male	25 (51.0%) 24 (49.0%)	71 (41.0%) 102 (59.0%)	0.697 0.697
Race - White - Asian - Black - Other	32 (65.3%) 1 (2.0%) 4 (8.2%) 12 (24.5%)	104 (60.1%) 18 (10.4%) 22 (12.7%) 29 (16.8%)	0.849 0.697 0.843 0.697
Ethnicity - Hispanic - Non-Hispanic - Other	9 (18.4%) 39 (79.6%) 1 (2.0%)	39 (22.5%) 127 (73.4%) 7 (4.0%)	0.849 0.843 0.849
Comorbidities - Asthma - Cancer - Chronic kidney disease - Chronic obstructive pulmonary disease - Obesity - Obstructive sleep apnea - Type 1 diabetes mellitus - Type 2 diabetes mellitus	5 (10.2%) 8 (16.3%) 9 (18.4%) 4 (8.2%) 7 (14.3%) 6 (12.2%) 1 (2.0%) 11 (22.4%)	9 (5.2%) 21 (12.1%) 15 (8.7%) 5 (2.9%) 16 (9.2%) 15 (8.7%) 2 (1.2%) 31 (17.9%)	0.476 0.536 0.271 0.333 0.536 0.536 0.536 0.536

For hospitalized post-clearance and non-hospitalized post-clearance cohorts, we considered phenotypes observed in clinical notes during three the following time periods as follows (i) Pre-COVID-19 (ii) SARS-CoV-2 positive (iii) Post viral clearance. The Pre-COVID-19 time period captures the phenotypes in the clinical notes in the year leading up to the SARS-CoV-2 infection. This period ends at Day −11 relative to the first positive PCR testing date to avoid capturing phenotypes that may be attributed to the SARS-CoV-2 infection. The second time period, SARS-CoV-2 positive, captures the phenotypes in the clinical notes which may have co-occurred with the SARS-CoV-2 viral infection. This period begins at Day −10 relative to the first positive PCR test, to account for the fact that most patients are infected with SARS-CoV-2 several days before their first positive PCR test. The final time period, Post viral clearance, captures the phenotypes in the clinical notes after the patient has cleared the SARS-CoV-2 viral infection. This period starts on the day after the estimated viral clearance date, which is the first of 2 or more consecutive negative PCR tests for the patient. This period extends until Day 90 relative to the estimated viral clearance date to capture phenotypes observed in the three months after the patient has cleared the SARS-CoV-2 virus from their system.

**Fig. 2:**
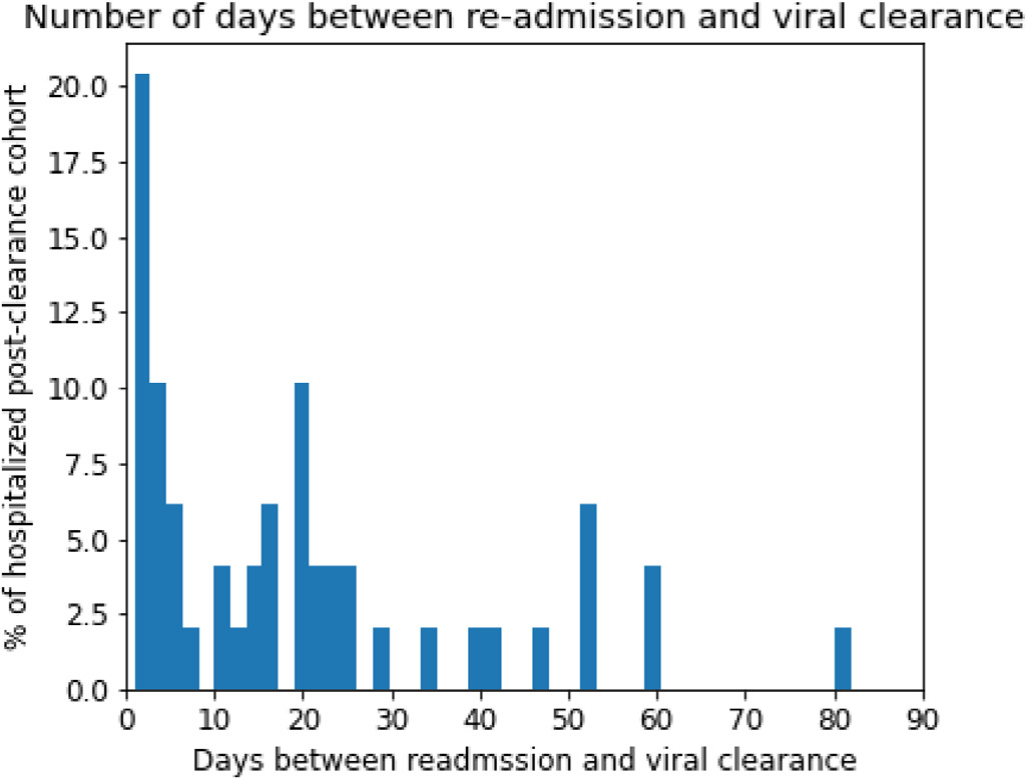
**Distribution of time to hospital readmission for post-clearance hospitalized cohort**. The x-axis corresponds to the number of days from the estimated viral clearance date to the date of hospital readmission post-clearance. The y-axis corresponds to the percentage of patients in the post-clearance hospitalized cohort.

We note that these time periods may vary for each patient depending upon the course of their SARS-CoV-2 infections. In particular, the Pre-COVID-19 and Post viral clearance time periods are always 355 and 90 days, respectively, however the SARS-CoV-2 positive time period depends upon the duration of the SARS-CoV-2 viral infection for each patient. In Fig. 2, we present the distribution of the number of days from viral clearance to re-hospitalization for the post-clearance hospitalized cohort.

As a note on data completeness, the demographics, hospital status, and relative clearance date covariates were available for all of the patients in the study population. For the phenotypes, which were derived from clinical notes, we assume that patients without clinical notes did not have any of the listed comorbidities or complications.

### 2.4 Augmented curation of clinical notes

A BERT-based neural network [20] was applied to identify phenotypes of interest in the clinical notes of the study population. The model was previously developed to classify the sentiment of general phenotypes [18] and thrombotic event phenotypes [21] in the encounter notes of COVID-19 patients. The categories of this classification model include: Yes (confirmed diagnosis), No (ruled out diagnosis), Maybe (possibility of disease), and Other (alternate context, e.g. family history of disease). This model was trained using nearly 250 different phenotypes and 18,500 sentences and achieves 93.6% overall accuracy and over 95% precision and recall for Yes/No sentiment classification [18]. For this study, the phenotypes of interest included a specific list of comorbidities and complications along with their synonyms. The comorbidities list consists of asthma, cancer, chronic kidney disease, chronic obstructive pulmonary disease, obesity, obstructive sleep apnea, type 1 diabetes mellitus, and type 2 diabetes mellitus. The complications list includes acute respiratory distress syndrome / acute lung injury, acute kidney injury, anemia, cardiac arrest, cardiac arrhythmias, disseminated intravascular coagulation, heart failure, hyperglycemia, hypertension, myocardial infarction, pleural effusion, pulmonary embolism, respiratory failure, sepsis, septic shock, stroke/cerebrovascular incident, venous thromboembolism / deep vein thrombosis

We ran the model to classify the sentiment for all of the above phenotypes in all of the clinical notes for each patient in the time periods considered in this study. Only sentences containing a phenotype with a positive sentiment (labeled “Yes” by the model) with a confidence of 0.95 or above were deemed positive mentions. Repeated sentences for the same patient are ignored. For each patient, in each time period we consider the phenotype to be present if there are at least 3 positive mentions in the clinical notes.

### 2.5 SARS-CoV-2 PCR tests conducted by mayo clinic hospitals and health system

In the context of PCR testing, patients seen at Mayo Clinic in Rochester MN were tested by either a laboratory-developed test or the Roche cobas SARS-CoV-2 assay [22,23]. Patients seen at Mayo Clinic's Florida hospitals and Arizona hospitals were tested using the Roche cobas test and Abbott diagnostic test respectively [24].

### 2.6 Statistical significance tests

Fisher's exact tests were applied pairwise across each of the baseline covariates (age, days to relative clearance, sex, race, ethnicity), comorbidities (refer to Table 1), and complications (refer to Table 2) in comparing the case and control cohorts, generating both a *p*-value and a relative risk value. These tests were applied using the SciPy package [25] in Python. The *p*-values were adjusted for multiple comparisons using the Benjamini-Hochberg (BH) correction. In cases where the relative risk cannot be computed because the incidence in the non-hospitalized cohort is zero, it is listed as “Undefined”.

**Table 2:** **Comparison of complications in the hospitalized and non-hospitalized post-clearance cohorts.** Complications in the hospitalized and non-hospitalized post-clearance cohorts, along with results from statistical significance tests stratified by time periods including: pre-COVID-19 infection, between SARS-CoV-2 infection and clearance, and post-COVID-19 infection. Phenotypes are identified in the physician notes via the neural network model with a minimum number of 3+ distinct mentions with a positive sentiment in the time horizon required to record a phenotype. Features which are significantly different between the two cohorts are highlighted in **green**. The columns are: **(1) Time window:** time window of interest, **(2) Phenotype:** phenotype of interest, **(3) Hospitalized post-clearance:** number of patients in the hospitalized post-clearance cohort with the phenotype recorded in the specified time horizon, **(4) Non-Hospitalized post-clearance:** number of patients in the non-hospitalized post-clearance cohort with the phenotype recorded in the specified time horizon, **(5) BH-corrected p-value:** Benjamani-Hochberg corrected p-values using Fisher's exact test for comparisons of proportions, **(6) Relative risk (95% CI):** Ratio of the phenotype incidence in the hospitalized post-clearance cohort to the phenotype incidence in the non-hospitalized post-clearance cohort, along with 95% confidence interval bounds. In cases where the relative risk cannot be computed because the incidence in the non-hospitalized cohort is zero, it is listed as “Undefined”.

Time Window	Phenotype	Hospitalized Post Clearance (49 patients)	Non-Hospitalized Post-Clearance (173 patients)	BH-corrected p-value	Relative risk (95% CI)
Pre-COVID-19	Acute kidney injury	7 (14.3%)	5 (2.9%)	0.030	4.9 (1.7, 13.6)
Pre-COVID-19	Acute Respiratory Distress Syndrome / Acute Lung Injury	1 (2.0%)	2 (1.2%)	0.769	1.8 (0.3, 15.4)
Pre-COVID-19	Anemia	13 (26.5%)	12 (6.9%)	0.007	3.8 (1.9, 7.6)
Pre-COVID-19	Cardiac arrhythmias	14 (28.6%)	17 (9.8%)	0.015	2.9 (1.5, 5.4)
Pre-COVID-19	Heart failure	7 (14.3%)	7 (4.0%)	0.066	3.5 (1.3, 9.1)
Pre-COVID-19	Hyperglycemia	1 (2.0%)	11 (6.4%)	0.755	0.3 (0.1, 2.4)
Pre-COVID-19	Hypertension	16 (32.7%)	46 (26.6%)	0.755	1.2 (0.8, 2.0)
Pre-COVID-19	Myocardial infarction	0 (0.0%)	3 (1.7%)	1.000	0.0 (0.0, 9.5)
Pre-COVID-19	Pleural effusion	3 (6.1%)	7 (4.0%)	0.755	1.5 (0.5, 5.6)
Pre-COVID-19	Pulmonary embolism	0 (0.0%)	6 (3.5%)	0.755	0.0 (0.0, 4.7)
Pre-COVID-19	Respiratory failure	1 (2.0%)	5 (2.9%)	1.000	0.7 (0.2, 5.6)
Pre-COVID-19	Sepsis	2 (4.1%)	4 (2.3%)	0.821	1.8 (0.4, 8.8)
Pre-COVID-19	Septic shock	1 (2.0%)	1 (0.6%)	0.755	3.5 (0.4, 32.7)
Pre-COVID-19	Stroke / Cerebrovascular incident	0 (0.0%)	1 (0.6%)	1.000	0.0 (0.0, 28.0)
Pre-COVID-19	Venous thromboembolism / Deep vein thrombosis	3 (6.1%)	4 (2.3%)	0.584	2.6 (0.7, 10.6)
SARS-CoV-2 positive phase	Acute kidney injury	7 (14.3%)	10 (5.8%)	0.318	2.5 (1.0, 6.0)
SARS-CoV-2 positive phase	Anemia	8 (16.3%)	11 (6.4%)	0.318	2.6 (1.1, 5.9)
SARS-CoV-2 positive phase	Acute Respiratory Distress Syndrome / Acute Lung Injury	10 (20.4%)	19 (11.0%)	0.318	1.9 (0.9, 3.7)
SARS-CoV-2 positive phase	Cardiac arrest	0 (0.0%)	1 (0.6%)	1.000	0.0 (0.0, 28.0)
SARS-CoV-2 positive phase	Cardiac arrhythmias	14 (28.6%)	29 (16.8%)	0.318	1.7 (1.0, 2.9)
SARS-CoV-2 positive phase	Heart failure	4 (8.2%)	7 (4.0%)	0.525	2.0 (0.7, 6.4)
SARS-CoV-2 positive phase	Hyperglycemia	5 (10.2%)	10 (5.8%)	0.525	1.8 (0.7, 4.9)
SARS-CoV-2 positive phase	Hypertension	11 (22.4%)	35 (20.2%)	0.795	1.1 (0.6, 2.0)
SARS-CoV-2 positive phase	Myocardial infarction	1 (2.0%)	1 (0.6%)	0.525	3.5 (0.4, 32.7)
SARS-CoV-2 positive phase	Pleural effusion	13 (26.5%)	12 (6.9%)	0.007	3.8 (1.9, 7.6)
SARS-CoV-2 positive phase	Pulmonary embolism	0 (0.0%)	4 (2.3%)	0.712	0.0 (0.0, 7.1)
SARS-CoV-2 positive phase	Respiratory failure	8 (16.3%)	16 (9.2%)	0.437	1.8 (0.8, 3.9)
SARS-CoV-2 positive phase	Sepsis	3 (6.1%)	5 (2.9%)	0.525	2.1 (0.6, 8.1)
SARS-CoV-2 positive phase	Septic shock	3 (6.1%)	3 (1.7%)	0.328	3.5 (0.8, 14.8)
SARS-CoV-2 positive phase	Venous thromboembolism / Deep vein thrombosis	0 (0.0%)	6 (3.5%)	0.525	0.0 (0.0, 4.7)
Post-viral clearance	Acute kidney injury	6 (12.2%)	1 (0.6%)	0.002	21.2 (2.6, 86.5)
Post-viral clearance	Acute Respiratory Distress Syndrome / Acute Lung Injury	6 (12.2%)	2 (1.2%)	0.006	10.6 (2.2, 37.6)
Post-viral clearance	Anemia	8 (16.3%)	1 (0.6%)	<0.001	28.2 (3.6, 108.7)
Post-viral clearance	Cardiac arrhythmias	4 (8.2%)	1 (0.6%)	0.021	14.1 (1.7, 64.5)
Post-viral clearance	Heart failure	2 (4.1%)	0 (0.0%)	0.067	Undefined (0.8, Infinity)
Post-viral clearance	Hyperglycemia	2 (4.1%)	0 (0.0%)	0.067	Undefined (0.8, Infinity)
Post-viral clearance	Hypertension	7 (14.3%)	5 (2.9%)	0.016	4.9 (1.7, 13.6)
Post-viral clearance	Myocardial infarction	1 (2.0%)	1 (0.6%)	0.424	3.5 (0.4, 32.7)
Post-viral clearance	Pleural effusion	11 (22.4%)	6 (3.5%)	<0.001	6.5 (2.5, 15.3)
Post-viral clearance	Respiratory failure	3 (6.1%)	1 (0.6%)	0.067	10.6 (1.2, 53.6)
Post-viral clearance	Sepsis	3 (6.1%)	2 (1.2%)	0.093	5.3 (1.0, 23.9)
Post-viral clearance	Septic shock	2 (4.1%)	1 (0.6%)	0.144	7.1 (0.8, 42.8)
Post-viral clearance	Venous thromboembolism / Deep vein thrombosis	2 (4.1%)	0 (0.0%)	0.067	Undefined (0.8, Infinity)

**Role of the funding source:** The funder was involved in the design and conduct of the study and data management. All the authors had access to the EHR records for all Mayo Clinic patients that were tested using a PCR based method for SARS-CoV-2. All authors approved the manuscript prior to publication.

## 3 Results

***COVID-19 patients hospitalized post-viral clearance have higher rates of pre-COVID-19 acute kidney injury, anemia, and cardiac arrhythmias as underlying conditions.***

In order to understand the patient characteristics that differentiated the ‘hospitalized post-clearance’ and ‘non-hospitalized post-clearance’ groups, we compared the clinical covariates in both the groups by analyzing phenotypes recorded with a positive sentiment in the patient history of both groups.

In Table 1, we present the demographics of the case and control cohorts, along with the comorbidities observed during pre-COVID-19 time period. For all of the demographic covariates, the cohorts are relatively well-balanced, and there are no statistically significant differences. Further, although the rates of comorbidities are elevated in the hospitalized post-clearance cohort, none of these differences are statistically significant.

In Table 2, we present the incidence of complications identified in the clinical notes from the (i) Pre-COVID-19 (ii) SARS-CoV-2 positive (iii) Post viral clearance time periods. For the pre-COVID-19 time period, we observe that three phenotypes are enriched in the hospitalized post-clearance cohort: (1) anemia (n: 13 (26.5%), relative risk: 3.8, 95% C.I.: [1.9,7.6], *p*-value: 0.007), (2) cardiac arrhythmias (n: 14 (28.6%), relative risk: 2.9, 95% C.I.: [1.5,5.4], *p*-value: 0.015), and (3) acute kidney injury (n: 7 (14.3%), relative risk: 4.9, 95% C.I.: [1.7,13.6], p-value: 0.030). We also note that these phenotypes are elevated in the hospitalized post-clearance cohort during the SARS-CoV-2 positive phase, however these differences during that time period were not statistically significant (p-values shown in Table 2). This suggests that for some clinical covariates, observations in the year leading up to SARS-CoV-2 infection may have more predictive power of post-clearance hospitalization than observations made exclusively during the index infection.

**Fig. 3:**
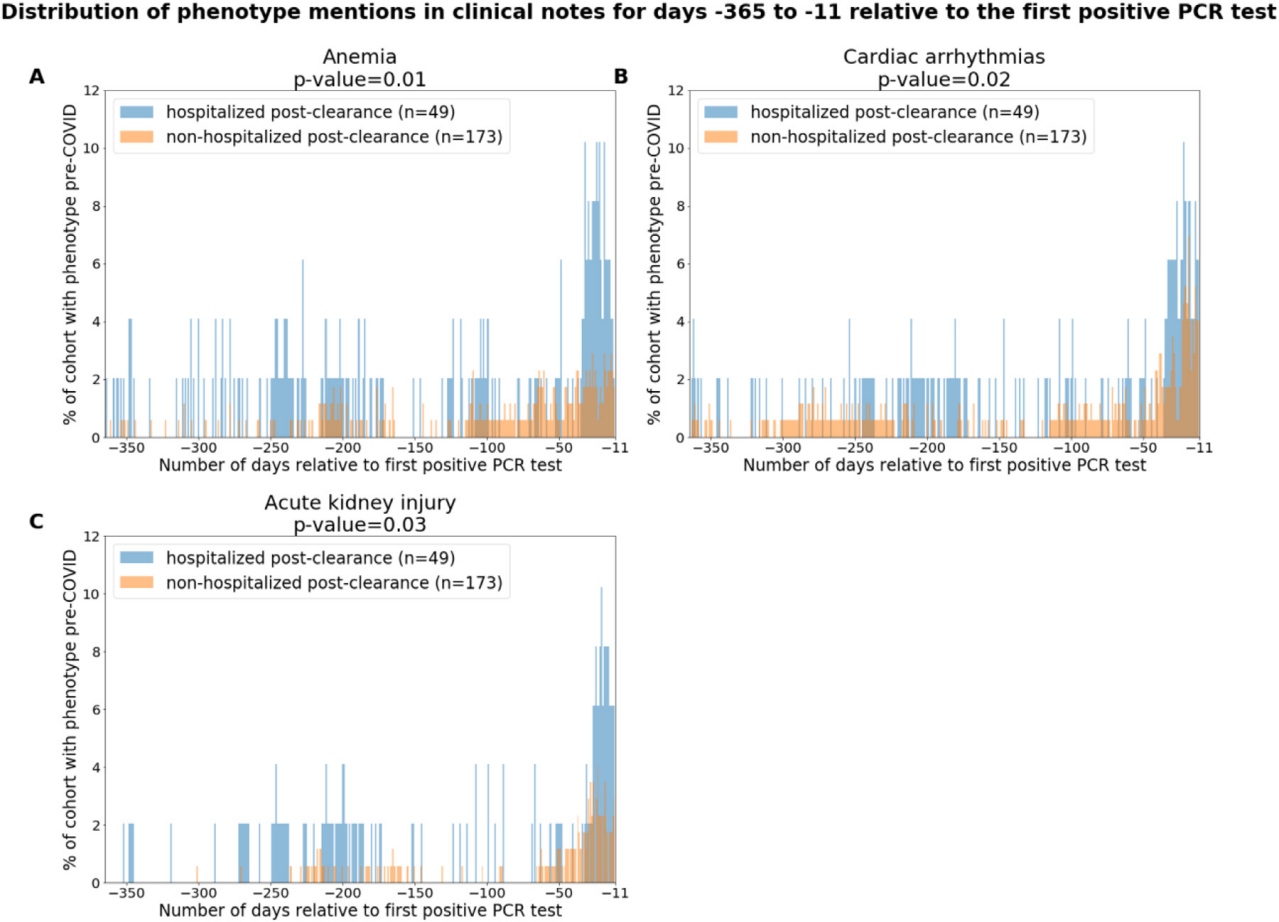
**Distributions of comorbidities in the hospitalized and non-hospitalized cohorts for days −365 to −11 relative to the first positive PCR test.** The phenotypes include: **(A)** Acute respiratory distress syndrome / acute lung injury, **(B)** Anemia, and **(C)** Cardiac arrhythmias. In the subplot for a single phenotype, the x-axis corresponds to the date relative to the first positive PCR date, and the y-axis corresponds to the percentage of patients with mentions of the phenotype with positive sentiment in their clinical notes on that relative date. In the title for each plot, we show the (phenotype, threshold for positive sentiment mentions) pair which is enriched in the pre-COVID-19 phase, along with the associated p-value. In the legend for each plot, we show the number and percentage of patients with the phenotype for the hospitalized post-clearance cohort in **blue** and for the non-hospitalized post-clearance cohort in **orange**.

In Fig. 3, we present the temporal distribution of phenotypes in the clinical notes for anemia, cardiac arrhythmias, and acute kidney injury for the pre-COVID-19 time period. We observe that both cohorts have higher rates of reports for all three phenotypes in the time period of −50 days to −10 days relative to the first positive PCR testing date. In addition, we observe that acute kidney injury is reported more frequently in the clinical notes from −200 days to −280 days relative to the first positive PCR testing date. These clusters of notes may reflect groups of patients who were admitted to the hospital with these phenotypes around the same time.

***Post-clearance of SARS-CoV-2 virus, COVID-19 patients experience complications including cardiovascular, renal, pulmonary, and immunological conditions***

In Table 2, we also include the rates of complications for the two cohorts of interest for the two time periods following PCR diagnosis of SARS-CoV-2: SARS-CoV-2 positive phase and post-clearance phase. For the COVID-19 post-clearance time period, we observe the following complications at higher rates in the hospitalized post-clearance cohort: acute kidney injury, anemia, acute respiratory distress syndrome / acute lung injury, anemia, cardiac arrhythmias, hypertension, pleural effusion. Among these, the most significantly enriched phenotypes are: anemia (relative risk: 28.2, 95% C.I.: [3.6,108.7], *p*-value < 0.001) and pleural effusion (relative risk: 6.5, 95% C.I.: [2.5,15.3], *p*-value < 0.001).

**Fig. 4:**
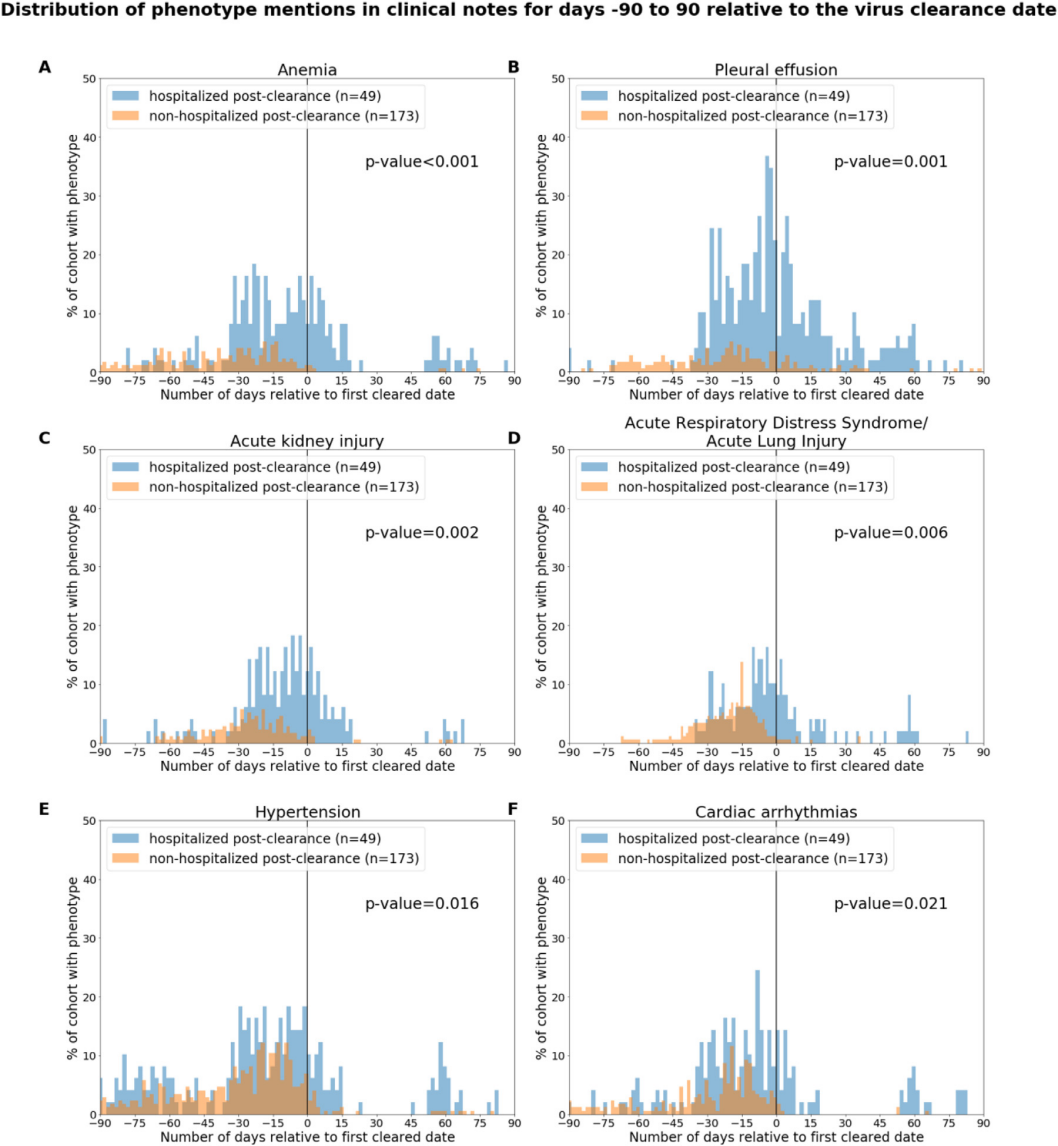
**Distributions of complications in the hospitalized and non-hospitalized cohorts for days −90 to 90 relative to the virus clearance date.** The phenotypes include: (A) Anemia, (B) Pleural effusion, (C) Acute kidney injury, (D) Acute respiratory distress syndrome/acute lung injury, (E) Hypertension, and (F) Cardiac arrhythmias. In the subplot for a single phenotype, the x-axis corresponds to the date relative to the virus clearance date, and the y-axis corresponds to the percentage of patients with mentions of the phenotype with positive sentiment in their clinical notes on that relative date. The clearance date (day = 0) is indicated by a vertical line. In the title for each plot, we show the phenotype which is enriched in the post-clearance phase, and in the right-hand side of each plot, we show the associated p-value. In the legend for each plot, we show the total number of patients in the hospitalized post-clearance cohort in **blue** and in the non-hospitalized post-clearance cohort in **orange**.

In Fig. 4, we show rates of these phenotypes over the +/- 90 days’ time window centered around the estimated viral clearance date for each patient. For most of these complications, we observe for the hospitalized post-clearance cohort that the majority of reports in the clinical notes occur within the first three weeks following the estimated viral clearance date. An exception is for pleural effusions, which occurs relatively frequently in the notes of the hospitalized post-clearance cohort in the two months following the clearance date. In addition, we observe moderate amounts of reports for anemia, cardiac arrhythmias, and hypertension around 50 to 70 days post-clearance.

## 4 Discussion

The topic of hospitalization and readmission in COVID-19 patients has been under major focus for mitigating national healthcare costs, particularly among 30-day readmissions that account for $17 billion in avoidable Medicare expenditures [26]. Prior studies have investigated hospitalization and readmission after recovery from other acute illnesses, such as influenza and community-acquired pneumonia [27], heart failure [28], and decompensated cirrhosis [29]. In the case of influenza, the most common cause of short-term readmission is due to non-influenza pneumonia [27]. These outcomes are associated with notably higher costs of care and increased mortality [27]. Given the widespread prevalence of SARS-CoV-2, similar outcomes warrant detailed studies in patients hospitalized after SARS-CoV-2 clearance.

Our analysis uses augmented curation methods to identify complications and comorbidities from the physician notes, rather than relying upon ICD codes. The statistical analysis identifies specific comorbidities in the year preceding PCR diagnosis of SARS-CoV-2 which are associated with increased rates of post-viral clearance hospitalization. We found that patients re-hospitalized after SARS-CoV-2 clearance had significantly higher odds of having pre-COVID-19 anemia, cardiac arrhythmias, and acute kidney injury compared to hospitalized controls. This finding may be useful for risk stratification of patients who have cleared the SARS-CoV-2 viral infection and have been discharged from the hospital. Furthermore, we observe that rates of re-hospitalization post-clearance are higher among patients who experience pleural effusions during their index infection. In addition, complications of acute kidney injury, acute respiratory distress syndrome / acute lung injury, anemia, cardiac arrhythmias, hypertension, and pleural effusions were also enriched in the hospitalized post-clearance cohort. These findings suggest that patients may be re-hospitalized post-clearance for a broad range of health conditions, including cardiac, respiratory, and renal conditions. One possible hypothesis is that many of these hospitalizations post-clearance were not due to reinfection per se, but rather secondary to a systemic inflammatory response.

There are several limitations of this study due to the observational nature of the data. First, we are not controlling for PCR testing frequency, so the study population which receives access to multiple PCR tests to confirm SARS-CoV-2 infection clearance may not be representative of the COVID-19 patient population in general. The phenotypes of complications and comorbidities which are identified in the notes with positive sentiment may include some false positives or false negatives based upon the neural network model predictions. In addition, the control groups for the hospitalized post-clearance cohorts were not propensity matched due to the limited cohort size. However, patients in the hospitalized cohort did not differ in demographic characteristics, and compared to prior investigations on this subject, the size of our cohort is a strength of the study. Next, the current time points chosen to align the patient journeys are anchored by the first positive PCR test and the first of the two or more negative tests. However the true viral clearance date for each patient is unknown. As an alternative study design, aligning the patient journeys based upon their hospitalization events could help identify the conditions that are enriched with respect to each hospitalization event. There is a limitation of the augmented curation model which is used to identify complications and comorbidities, because it only determines whether or not the phenotype was mentioned with positive sentiment in a clinical note, and does not take into account the temporal component. Therefore, although we know that certain phenotypes are enriched in the cohorts which are admitted to the hospital/ICU post-clearance, the dates of these complications are uncertain. There is research ongoing to develop natural language processing based neural network models which can differentiate effectively between active and historical phenotype diagnoses [30], which could strengthen these conclusions for future analyses. Finally, we note that this study was from a single health system, so the underlying clinical characteristics of the study population are biased to reflect the clinical characteristics of individuals that receive medical treatment in certain regions of the United States (Arizona, Florida, Minnesota). A follow-up study will be required to generalize the findings to a broader population.

The phenotypes described here relate to EHR based mentions of conditions. Future work would include a complementary analysis based on lab tests relevant to the identified phenotypes such as estimated Glomerular Filtration Rate (eGFR), Blood Urea Nitrogen test, Hemoglobin, Hematocrit, Alanine transaminase (ALT), Aspartate transaminase (AST), and Bilirubin. A longitudinal analysis integrating clinical notes-based augmented curation and lab-measurements will be useful in formulating a case definition of long-term COVID-19 (long-COVID). Overall, our findings motivate additional studies to explore the biological mechanisms of SARS-CoV-2 driven long-term adverse effects in order to find appropriate prophylactic and therapeutic interventions. This study also emphasizes the need for detailed curation of structured and unstructured clinical data to better understand the dynamics of viral clearance, underlying conditions, and long-term complications.

## Author contributions

CP and AV contributed to the study design, project supervision, and writing. ER and CK performed the formal analysis and contributed to the writing. AP, NK, and GB contributed to the statistical analysis and validated the augmented curation models. AA and RB conceptualized and trained the augmented models, and RB contributed to the writing (review and editing). JO and ADB contributed to the investigation and writing (review and editing). VS conceived the study design, contributed to the investigation, project administration, project supervision, and writing (review and editing). All authors approved the manuscript prior to publication.

## Data sharing statement

After publication, the data will be made available to others upon reasonable requests to the corresponding author. A proposal with detailed description of study objectives and statistical analysis plan will be needed for evaluation of the reasonability of requests. Deidentified data will be provided after approval from the corresponding author and the Mayo Clinic's standard IRB process for such requests.

## Funding

This work was supported by Nference, inc.

## Declaration of Competing Interest

CP, AJV, ER, CK, GB, AP, NK, AA, RB, ADB, and VS have financial interests in nference, Inc. ADB is a consultant for Abbvie and Flambeau Diagnostics and is a DSMB member at Corvus and Equilium. ADB is on the scientific advisory boards for nference and Zentalis and is founder and President of Splissen therapeutics. JCO receives personal fees from Elsevier and Bates College, and receives small grants from nference, Inc, outside the submitted work. CP, AJV, ER, CK, GB, AP, NK, AA, RB and VS are nference employees. CP, NK, VS, CK, AA, AP, AJV, ER, GB and RB received personal fees from nference. The Mayo Clinic has a Financial Conflict of Interest in technology used in the research and may stand to gain financially from the successful outcome of the research. This research has been reviewed by the Mayo Clinic Conflict of Interest Review Board and is being conducted in compliance with Mayo Clinic Conflict of Interest policies.
